# A neuroscientist’s guide to neural burst detection

**DOI:** 10.1162/IMAG.a.1226

**Published:** 2026-04-30

**Authors:** Lindsey Power, Idil Aydin, Timothy Bardouille, Sylvain Baillet

**Affiliations:** McConnell Brain Imaging Centre, Montreal Neurological Institute, McGill University, Montreal, Quebec, Canada; Department of Physics & Atmospheric Science, Dalhousie University, Halifax, Nova Scotia, Canada; Centre de recherche du Centre Hospitalier de l’Université de Montréal (CRCHUM), Montreal, Quebec, Canada; Department of Neuroscience, Université de Montréal, Montreal, Quebec, Canada

**Keywords:** neural bursts, electrophysiology, transient events, methods, tutorial

## Abstract

Neural bursts—brief episodes of heightened oscillatory activity—are increasingly studied as fundamental building blocks of brain function, with relevance to perception, cognition, and disease. As such, detecting and characterizing these bursts in electrophysiological recordings have driven rapid methodological innovation in neuroscience research. However, the growing number of analysis techniques can be overwhelming, making it difficult for researchers to select the most appropriate method for their specific goals. In this review, we provide a structured and practical guide for neuroscientists to measure and interpret neural burst data. We offer an overview of current detection methods, accompanied by a suite of tutorials, including code notebooks and data, to enable concrete implementation and critical evaluation. We then conclude with actionable recommendations to help researchers select the best burst detection strategy in diverse research contexts. This guide is intended for newcomers to the field as well as more experienced neuroscientists seeking to expand their methodological toolkit.

## 1 Introduction

Neural bursts are characterized by brief episodes of high-power electrophysiological activity. They have been observed across varied behaviors in multiple species, brain regions, using varied recording modalities, and are increasingly recognized as hallmarks of brain function. These transient events are associated with a wide range of processes including sensory encoding, motor control, arousal, and cognition. Despite their neuroscientific significance and increasing prevalence in the literature, the detection and interpretation of transient neural bursts in electrophysiological signals of growing complexity remain challenging and lack standardized analytic procedures.

Studies of brain circuit dynamics have traditionally emphasized amplitude modulations of neural oscillations induced by external events, such as sensory stimuli, or those time-locked to behavioral changes, such as motor outputs. However, recent work (Jones, 2016; van Ede et al., 2018) has emphasized that macroscopic electrophysiological recordings, such as from intracranial local field potentials (LFP) or non-invasive electro- and magnetoencephalography (EEG/MEG), feature endogenous, transient oscillatory signal elements (see Box 1). These *bursts* are commonly defined by their short duration, elevated power relative to background activity, and a narrow frequency bandwidth. However, these defining features vary across studies depending on the detection method and research context.

Box 1.  Cellular Mechanisms of Neural Bursts

Rhythmic activity in the cortex emerges from frequency-specific synchronization of neuronal populations (Lopes da Silva, 1991), with signal power determined by the number and coherence of the neurons involved (Murthy & Fetz, 1996). Since Hans Berger’s first observations of alpha rhythms (8–12 Hz; Berger, 1929; Mushtaq et al., 2024), a range of complementary techniques—including EEG, MEG, and local field potential (LFP) recordings to measure large-scale activity, single-unit electrophysiology to track spike timing, and *in vivo* and *in vitro* imaging to interrogate circuit-level processes—have been used to study neural oscillations. These experimental approaches are complemented by computational models of neural network dynamics (Wang, 2010).

The collective findings point to a complex interplay between intrinsic cellular properties and network-level architecture in the generation of rhythmic neural activity. At the cellular level, intrinsic oscillatory properties of neurons (Buskila et al., 2019; Wang, 2010), regulated by ion channel kinetics (Kadala et al., 2015) and modulated by astrocytic signaling (Bellot-Saez et al., 2017; Buskila et al., 2019), shape the propensity for rhythmic firing. At the network level, synchronous activity is orchestrated through both chemical and electrical synapses (Timofeev et al., 2012; Wang, 2010), as well as through structured thalamocortical interactions that facilitate local and long-range coordination (Steriade, 2004).

Historically, electrophysiological studies often interpreted neural oscillations as prolonged, tonic phenomena—a view shaped in part by trial averaging. However, it is now more widely recognized that such oscillations may be constituted by brief, intermittent bursts whose occurrences may jitter on a trial-by-trial basis, thereby producing the illusion of tonic oscillatory power changes following trial averaging (Jones, 2016). This shift in perspective has motivated advances in computational modeling (e.g., Sherman et al., 2016) to better account for the transient and nonstationary nature of neural activity.

Analyzing ongoing or event-related neural activity at the level of discrete bursts, defined by properties such as peak frequency, peak power, duration, and rate of occurrence, has revealed mechanistic insights often obscured by more traditional analyses. For instance, bursts with greater peak power suggest the transient recruitment of larger or more coherent neural ensembles (Murthy & Fetz, 1996), while increased burst rate or duration may indicate changes in local excitability or broader shifts in network state (Suresh et al., 2016). These features provide an expanded set of candidate markers of physiological function and dysfunction, deepening our understanding of both healthy and pathological brain dynamics.

Neural bursts have long been linked to numerous cognitive and behavioral functions, including attention (Lakatos et al., 2004; Vigué-Guix & Soto-Faraco, 2023), working memory (Lundqvist et al., 2016, 2022, 2024), arousal and relaxation (Cantero & Atienza, 2000; Hebert & Lehmann, 1977), and voluntary movement (Errington et al., 2020; Feingold et al., 2015; He et al., 2020; Little et al., 2019; Wessel, 2020; West et al., 2023). More recently, they have been shown to be signal constituents of what traditional analyses had measured as sustained rhythmic activity (Jones, 2016). Certain burst features, particularly in the beta band (15–30 Hz), evolve with development and aging (Brady et al., 2020; Power & Bardouille, 2021; Rayson et al., 2023), and are affected by neurological conditions, including Parkinson’s disease (Lofredi et al., 2018; Tinkhauser et al., 2017; Vinding et al., 2020) and schizophrenia (Briley et al., 2021; Spencer et al., 2023). As such, neural bursts also hold promise as potential clinical biomarkers.

Methodologically, bursts have been traditionally analyzed on the basis of relatively simple characteristics, such as rate of occurrence, burst duration, or peak amplitude. More recently, waveform features of bursts, such as their shape, sharpness, and rise–decay asymmetry, have been discussed as functionally relevant to both cognitive (Lundqvist et al., 2024; Szul et al., 2023) and disease (Cole & Voytek, 2017) states. Such proliferation of detection techniques, grounded in different assumptions and analytical purposes, with varying computational demands, challenges the reproducibility of neuroscience research and the interpretability of neural burst effects.

The present review aims to demystify burst detection by providing a structured, accessible user guide for adopting the most appropriate approach for specific research questions. We begin with a high-level overview of the most widely used burst detection methods, outlining their theoretical foundations, practical implementations, and pragmatic trade-offs. We then provide practical tutorials and worked examples to illustrate the respective performance of the featured methods. Finally, we present a decision-making framework to help researchers align method selection with their research question and experimental design.

## 2 Overview of Burst Detection Methods

**Fig. 1.**
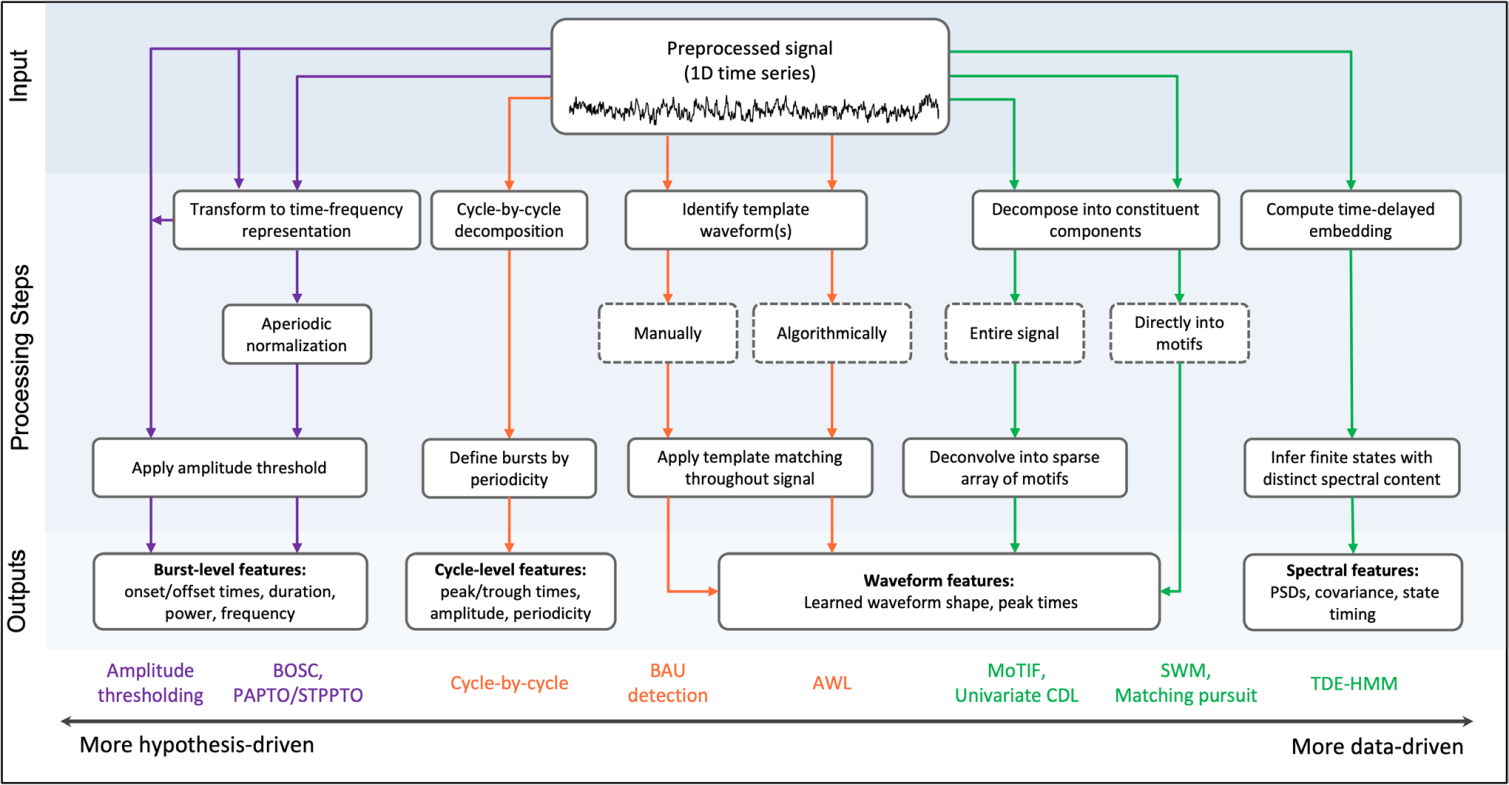
Overview of single-channel burst detection methods. Flowchart illustrating the input (top row), processing steps (middle rows), and outputs (bottom row) for single-channel burst detection methods, listed as colored text labels at the bottom of the chart. Methods are arranged from left to right along a gradient from *more hypothesis-driven* to *more data-driven*, reflecting their respective requirements for a priori assumptions. Arrows and method names are colored according to their method class (purple: amplitude-based, orange: waveform-based, and green: motif-based). Dashed contour boxes indicate variants of the prior step that distinguish between methods. All approaches require a preprocessed input signal (e.g., artifact removal and quality control; Jas et al., 2018; Tadel et al., 2019), with optional frequency filtering to restrict burst extraction to a predefined band of interest (e.g., 15–30 Hz for beta bursts). Additional post hoc selection criteria can be applied to retain only bursts exceeding a minimum duration (e.g., >100 ms). This schematic emphasizes differences in how bursts are defined, the algorithmic implementations used, and the model outputs produced.

In this section, we review commonly used burst detection methods along a continuum from hypothesis-driven to data-driven approaches (Fig. 1). These methods differ in how they define bursts, the prior assumptions they make about signal characteristics, and the nature of their outputs. Further, methods may be designed to detect bursts at a single “channel” (i.e., implanted electrode, scalp EEG, or MEG sensor), or across multiple spatially distributed channels.

In this review, methods are divided into three classes: **amplitude-based methods, waveform-based methods, and motif-based methods**. The majority of the methods discussed in this review operate on single-channel time series, with the exception of a few motif-based approaches that have been proposed for more complex multichannel burst detection. An overview of typical applications and implementation resources for each method is given in Table 1.

**Table 1.** Overview of resources for burst detection.

METHOD	Key Parameters	Applications	Implementation Resources	Notes
Amplitude Thresholding	Power threshold, frequency transform parameters, optional duration threshold	Widely used for detecting bursts in sensorimotor beta, alpha, and gamma bands (e.g., Shin et al., 2017)	Available in MATLAB and Python https://github.com/jonescompneurolab/SpectralEvents	Simple, hypothesis driven; best for known frequency bands
eBOSC	Power threshold, duration threshold, wavelet parameters	Detection of delta, theta, alpha, and beta bursts in scalp EEG and MEG (e.g., Kosciessa et al., 2020; Seymour et al., 2022)	Available in MATLAB https://github.com/jkosciessa/eBOSC and Python https://github.com/jkosciessa/eBOSC_py	Models aperiodic background; computationally intensive
PAPTO	Power threshold, wavelet parameters, specparam parameters	Sensorimotor beta burst detection in MEG (Brady & Bardouille, 2022)	Available in Python https://github.com/tbardouille/papto_camcan	Models aperiodic background; improved sensitivity with specparam-based normalization
STPPTO	Power threshold, superlet parameters, specparam parameters	Alpha and beta burst detection in MEG (Liang et al., 2024)	Not publicly available	Time-resolved specparam fitting; high time–frequency resolution
BAU Detection	Template definition	Detection of task-related sharp-wave components in single-unit recordings (Abeles, 2014)	Not publicly available	Template based; limited generalizability; used mainly in single-unit data
AWL	Template definition, adaptability parameters	Detection of repeating spike-like motifs (Hitziger et al., 2017)	Available in C++ and MATLAB https://github.com/hitziger/AWL	Reduced template rigidity; risk of overfitting
Cycle-by-cycle	Cycle consistency parameters, minimum cycles	Applied to hippocampal and sensorimotor rhythms (Cole & Voytek, 2019; Gunasekaran et al., 2023)	Available in MATLAB https://github.com/StefanoBuccelli/bycycle_matlab and Python (bycycle package) https://github.com/voytekresearch/Cole_2018_cyclebycycle	Waveform-shape based; good for analyzing rhythmic structure
MoTIF	Atom length, number of atoms	Detection of repeating spike-like motifs (Hitziger et al., 2017)	Available in MATLAB https://github.com/hitziger/AWL	Identifies diverse, de-correlated burst patterns
Sliding Window Matching	Window length, number of windows, number of motifs	Applied to gamma and theta bursts in LFP (Gips et al., 2017)	Available in MATLAB https://github.com/bartgips/SWM	Captures recurring motifs; parameter sensitive
Matching Pursuit	Initial atom definition	Detection of gamma bursts in primate LFP recordings (Chandran et al., 2018)	Available in MATLAB https://github.com/supratimray/GammaLengthProjectCodes	Identifies diverse waveforms; computationally intensive
HMM	Number of embeddings (TDE) number of PCA components, number of states	Detection of alpha and beta bursts in real and simulated data (Quinn et al., 2019)	Available in MATLAB https://github.com/OHBA-analysis/HMM-MAR and Python (osl-dynamics package) https://github.com/OHBA-analysis/osl-dynamics	Data driven; detects multiple spectrally distinct events simultaneously
CDL	Atom length, number of atoms	Applied to detect alpha, beta, and gamma bursts in MEG and scalp EEG (Power et al., 2023; Wang et al., 2024)	Available in Python (alphacsc package) https://alphacsc.github.io/stable/index.html	Detects waveform “atoms” across time and space (optional)

### 2.1 Amplitude-based methods

Amplitude-based approaches tend to be largely hypothesis driven, defining bursts using predetermined criteria based on amplitude thresholds or spectral features aligned with prior expectations.

The most widely used example is simple **amplitude thresholding**, where bursts are identified as transient amplitude increases within a target frequency band (for example, 15–30 Hz for beta bursts). After band-pass filtering, local amplitude maxima are extracted either directly from the time series (e.g., Gómez et al., 2022; Tinkhauser et al., 2017) or from a time–frequency representation of the data (e.g., Brady & Bardouille, 2022; Feingold et al., 2015; Lundqvist et al., 2016; Shin et al., 2017). Thresholds are often set as a multiple of the median or a specific percentile of the amplitude distribution (e.g., >75th or 90th percentile). Minimum duration criteria (e.g., >3 cycles or >100 ms) are also sometimes applied to further filter out spurious events. Although conceptually simple and widely applicable, this approach can misclassify noise or sustained oscillations (such as hippocampal theta at 4–8 Hz; Cole & Voytek, 2019) as bursts, and it is less effective when background activity is non-stationary or strongly aperiodic.

To address some of these limitations, the **Better OSCillation detection (BOSC)** method (Caplan et al., 2001; Hughes et al., 2012; Whitten et al., 2011) incorporates theoretical modeling of the aperiodic 1/f spectrum (Donoghue et al., 2020). The original BOSC approach uses a fixed linear background model and assumed stationarity over time and detects bursts when both a power threshold and a duration threshold (e.g., three cycles) are exceeded. An enhanced version of this approach, **extended BOSC (eBOSC**; Kosciessa et al., 2020), improves background estimation by using robust regression in log–log space. This adaptation better accounts for local variations in background activity, reducing false positives in data with non-stationary noise or prominent 1/f components.

Another adaptation of amplitude thresholding, **Periodic/Aperiodic Parametrization of Transient Oscillations (PAPTO**; Brady & Bardouille, 2022), first removes the modeled aperiodic background using the *specparam* algorithm (Donoghue et al., 2020), which can accommodate linear and non-linear spectral shapes (including “knees”). Burst detection is then applied to the reconstructed periodic component using the same peak-based procedure as in amplitude thresholding.

Finally, **Super-resolution Time**–**frequency Periodic Parameterization of Transient Oscillations (STPPTO**; Liang et al., 2024) further builds upon these methods by calculating time-resolved aperiodic estimations prior to burst detection. The method combines *specparam* with the *superlet* transform (Moca et al., 2021), calculating the aperiodic component at each time point to achieve high-resolution time–frequency analysis. This approach allows dynamic background modeling that adapts to rapid spectral changes, enabling more reliable detection of bursts under highly non-stationary conditions (Schaworonkow & Voytek, 2021; Wilson et al., 2022). As with other highly parameterized approaches, STPPTO trades generality for specificity, and its performance depends critically on the stability of time-resolved aperiodic fits.

Overall, amplitude-based methods offer transparent, interpretable thresholds and are well suited for testing targeted predictions about bursts in specific frequency bands. However, these methods rely on the accuracy of prior assumptions about signal structure—such as predefined burst frequency bands, minimum duration thresholds, and canonical waveform shapes—that may limit their ability to fully capture the complexity and variability inherent of neural signals.

### 2.2 Waveform-based methods

In contrast, waveform-based methods define bursts from the morphological properties of the data itself, providing a more data-driven approach to burst identification. This makes them useful for detecting non-sinusoidal waveforms or recurrent patterns that may not fit narrow a priori definitions (Cole & Voytek, 2017; Donoghue et al., 2022; van Dijk et al., 2010). A growing body of evidence suggests that waveform diversity (e.g., in terms of sharpness asymmetry, rise–decay asymmetry) reflects distinct underlying circuit mechanisms rather than mere noise (Rayson et al., 2026). Thus, investigating waveform properties of bursts instead of assuming canonical shapes may provide additional mechanistic insights.

One such technique, **cycle-by-cycle** analysis, decomposes the signal into individual oscillatory cycles defined by peaks, troughs, and zero-crossings (Cole & Voytek, 2019). Each cycle is characterized by amplitude, period, and symmetry, and those meeting consistency criteria (such as monotonic rise/fall and stable amplitude) are classified as burst-like. This method is effective for isolating non-sinusoidal or irregular rhythms, though it may struggle when faster oscillations overlap in time.

Template matching offers another series of approaches, comparing short, overlapping windows of the signal to a reference waveform template that is either manually selected or generated algorithmically. Similarity is assessed through sliding-window correlation or convolution filtering. For example, **Brief Amplitude Undulation (BAU)** detection uses manually identified representative waveforms from the data to identify recurring fluctuations (Abeles, 2014). While highly specific to the chosen morphology, this type of template matching is sensitive to bias in template selection. **Adaptive Waveform Learning (AWL)** builds on template matching by iteratively updating templates using algorithms such as matching pursuit or expectation–maximization (Hitziger et al., 2017). These refinements allow for time warping and better capture of within-subject or between-subject variability in burst shape, though careful regularization is needed to avoid overfitting.

Waveform-based methods provide a flexible approach to burst detection that reduces biases imposed by traditional frequency transforms and allows for the realization of diverse waveform shapes. However, these methods still rely on a number of user-defined parameters that can significantly impact the model outputs.

### 2.3 Motif-based methods

Motif-based methods provide a more strictly data-driven approach, decomposing the signal into sets of basis waveforms (“atoms” or “motifs”) that can be linearly combined to reconstruct the signal. Unlike template matching, these methods do not require the user to specify predefined waveform shapes, making them highly flexible options for burst detection. However, practical implementations often constrain the search space to reduce computational cost or target specific frequency ranges. As a result, these methods can miss rare but physiologically relevant events if the algorithm focuses on the most dominant motif. Pre-processing steps such as component separation (see Box 2) can help highlight motifs of interest before motif estimation.

Box 2.  Signal Decomposition Methods

Signal decomposition methods transform single- or multi-channel recordings into a set of distinct component time series, each reflecting a specific feature of the original signal according to predefined mathematical criteria. Although these methods can greatly aid exploratory analyses or preprocessing, they are not designed to detect bursts directly. Instead, they generate continuous component signals from which bursts can later be identified using separate detection algorithms.

In single-channel recordings, techniques such as **Empirical Mode Decomposition** (**EMD**; Huang et al., 1998) and its refinements, including **iterated masking-EMD** (Fabus et al., 2021), partition the signal into a finite set of intrinsic mode functions (IMFs) spanning the full recording duration. EMD offers a data-driven alternative to traditional band-pass filtering, allowing physiologically meaningful oscillatory modes to emerge from the data without fixed basis functions. These modes can then be examined with standard burst detection algorithms to identify transient events and characterize their timing, duration, and morphology (Quinn et al., 2021).

Multi-channel recordings often require decomposition methods capable of separating overlapping neural sources and isolating oscillations from artifacts. **Principal Component Analysis (PCA)** and **Independent Component Analysis (ICA)** achieve this by modeling the data as weighted linear mixtures of latent sources (Vigário et al., 1998). PCA identifies orthogonal components that explain the largest proportion of variance, whereas ICA further imposes statistical independence among components (Hyvärinen & Oja, 2000). In burst research, these techniques have been used to extract components that closely match rhythms of interest, such as sensorimotor beta activity, before applying single-channel burst detection (Briley et al., 2021). PCA has also been applied after burst detection to group similar waveforms into clusters, enabling the study of variability in burst shape and structure (Quinn et al., 2021; Szul et al., 2023).

Despite their utility, EMD, PCA, and ICA have limitations for burst analysis. They produce continuous time series rather than discrete event labels, making it necessary to combine them with additional detection steps. Moreover, ICA’s assumption of statistical independence can be problematic when neural processes are functionally coupled or overlap in space, for example, in co-occurring beta and mu rhythms from the sensorimotor cortex. Nevertheless, when applied judiciously, signal decomposition methods can improve the sensitivity and specificity of burst detection pipelines, enhance interpretability by separating neural and artifactual contributions, and provide a principled basis for examining oscillatory components in complex datasets. An illustrative example of how signal decomposition methods can be integrated into the burst detection workflow is provided below.

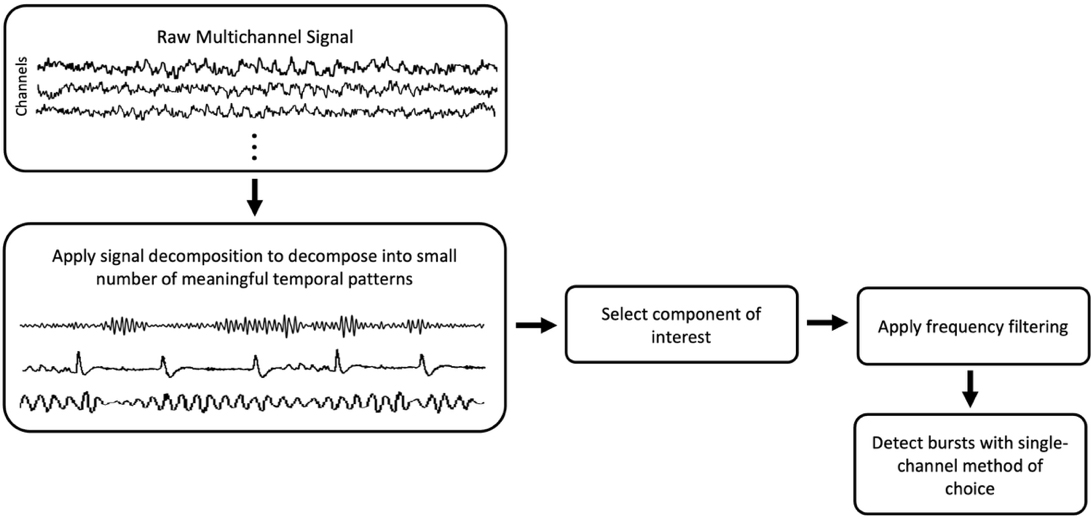

Several algorithms fall into this category. Univariate **Convolutional Dictionary Learning (CDL**; sometimes referred to as convolutional sparse coding or CSC) represents the signal as a sparse sum of unique temporal “atoms” convolved with their activation time courses, estimated through coordinate descent (Jas et al., 2017). **Sliding Window Matching (SWM**; Gips et al., 2017) uses a Monte Carlo-based stochastic search to find recurring segments, with detection sensitivity influenced by window length and spacing. **Matching of Time-Invariant Filters (MoTIF**; Jost et al., 2006) adds a decorrelation penalty to encourage diversity in the extracted motifs, potentially sacrificing detection of dominant but redundant patterns. **Matching Pursuit (MP**; Chandran et al., 2016, 2018; Locatello et al., 2018) instead works with an overcomplete dictionary, iteratively selecting the motif that best reduces reconstruction error.

A related algorithm, **Hidden Markov Modelling (HMM)**, models the data as being generated by a finite set of hidden states, each associated with a probability distribution (Baker et al., 2014). The model produces a time series of state probabilities, which can be thresholded to identify discrete “on” and “off” periods. Two extensions of HMM, **amplitude-envelope (AE)-HMM** and **time-delay embedded (TDE)-HMM** have been validated as methods of single-channel burst detection (Quinn et al., 2019). AE-HMM is applied to the amplitude envelope of a bandpass filtered signal, segmenting the data into two states: a high-power burst state and a lower power non-burst state, providing information on timing of high-power events. TDE-HMM, however, captures states with distinct spectral properties allowing for the identification of a user-defined number of states, each representing a spectrally distinct burst type. A limitation, however, is that HMM assumes mutual exclusivity, only one state can be active at a given time, restricting its capacity to model overlapping burst types. Importantly, these single-channel HMM formulations detect statistical state structure rather than bursts per se, and their interpretation as burst detectors depends on strong alignment between state structure and focal transient events.

### 2.4 Multichannel motif-based methods

Although most burst detection studies have relied on single-channel approaches, some research questions demand the integration of spatial information to fully capture the dynamics of neural activity. This becomes essential when the anatomical origin of bursting is uncertain or when there is interest in multiple, spatially distinct burst types. In such contexts, treating bursts as isolated events can obscure important spatiotemporal and topographical aspects of the data. Multi-channel burst detection methods address this limitation by characterizing bursts across multiple channels simultaneously, providing a richer view of their spatial organization.

Extending the univariate CDL approach, **Multivariate Convolutional Dictionary Learning (CDL)** exemplifies a powerful approach for discovering short, repeated temporal patterns (typically lasting 100–1000 ms) in multi-channel datasets, along with their corresponding spatial topographies (Dupré la Tour et al., 2018). Unlike ICA, which assumes statistical independence among sources (see Box 2), CDL assumes that repeated waveform motifs exist in the data and can optionally incorporate spatial smoothness constraints to mitigate volume conduction effects in M/EEG. Because CDL does not impose mutual exclusivity between atoms, it can detect overlapping bursts arising from different networks. Its performance depends on user-specified parameters such as the number and expected duration of atoms, which must be tuned to match the temporal scale of the bursts under study. When these parameters are chosen appropriately, CDL has shown strong interpretability and effectiveness in both task-evoked and spontaneous burst analyses (Power et al., 2023; Wang et al., 2024).

TDE-HMM is another motif-based framework that may be adapted for burst-like applications. Multichannel TDE-HMM has been established in functional connectivity analysis for segmenting time-varying brain activity into discrete, transient “brain states” with distinct spectral properties (Vidaurre et al., 2018). While such spatially and spectrally resolved transient states may be related to multichannel bursting events, this is likely only true in cases where states reflect relatively focal spatial patterns. In contrast, states that map to broad, spatially distinct cortical areas likely reflect large-scale network activity not directly related to neural bursting. Further work is required to adapt TDE-HMM for the specific goal of detecting focal bursting events across multiple channels simultaneously.

In general, motif-based approaches are highly data driven and can reveal complex, non-sinusoidal burst structures that hypothesis-driven methods might miss. However, they often demand greater computational resources and can be more challenging to interpret than simpler amplitude or waveform-based approaches.

### 2.5 Summary

Neural burst detection methods span a continuum from hypothesis-driven strategies, which rely on predefined thresholds and assumptions about oscillatory activity, to data-driven strategies, which learn burst characteristics directly from the data. Hypothesis-driven approaches are simple, interpretable, and well suited to targeted questions, while data-driven approaches are flexible and better equipped to capture complex or atypical burst morphologies. The choice of method depends on the working definition of a burst, the level of interpretability required, and the computational resources available. Signal decomposition tools (Box 2) can complement, but not replace, these detection methods, and their integration into the analysis pipeline should be guided by the research objectives.

## 3 Tutorials

This section walks through practical, step-by-step examples for seven widely used burst detection methods: amplitude thresholding, PAPTO, cycle-by-cycle analysis, eBOSC, sliding window matching, time delay embedded hidden Markov modeling (TDE-HMM), and multivariate convolutional dictionary learning (CDL). The aim is to provide an applied reference that researchers can follow directly or adapt to suit different datasets, preprocessing strategies, and software environments. These tutorials are intended to provide basic examples of method implementation, with parameter selection based on common practice in the literature. If repurposing this code for research applications, users should consult method-specific literature to ensure the selected parameters are appropriate for the given application and that all model assumptions (aperiodic fit, model convergence, etc.) are satisfied. All annotated scripts, datasets, and helper functions are freely available at https://github.com/lindseypower/BurstDetection_Tutorials, with method-specific resources and references summarized in Table 1.

All examples are based on 10 minutes of resting-state, eyes-open MEG data from the Open MEG Archive (OMEGA) (Niso et al., 2016), formatted according to MEG-BIDS (Niso et al., 2018) and available on OpenNeuro (dataset ds000247, version 1.0.2, participant sub-0002). Data were acquired with a 275-channel whole-head CTF MEG system (VSM MedTech Inc., Canada) at 2400 Hz, using an anti-aliasing low-pass filter at 600 Hz. Digitized landmarks and head shape were recorded for anatomical coregistration. EOG and ECG traces accompanied the MEG data, and each participant underwent high-resolution T1-weighted MRI for source modeling and cortical parcellation with *FreeSurfer* v5.3 (Fischl, 2012).

Preprocessing, performed in *Brainstorm* (Tadel et al., 2011), included coregistration to the individual MRI, band-pass filtering between 1 and 50 Hz, downsampling to 250 Hz, and artifact attenuation by signal-space projection (SSP) for blinks and cardiac activity. The forward model was computed with the overlapping-sphere approach (Huang et al., 1999), and sources were reconstructed using dSPM (Dale et al., 2000) with a noise covariance matrix derived from empty-room recordings. Cortical time series were parcellated with the Schaefer-200 atlas (Schaefer et al., 2018). A complete *Brainstorm* preprocessing tutorial is provided in the tutorial repository.

Single-channel examples were extracted by averaging polarity-corrected source activity from the right primary motor cortex (parcel *SomMotA_4 R* in Schaefer-200). Single-channel data were then filtered to the beta band (15–30 Hz). Bursts were detected from the extracted one-dimensional time series using six standalone burst detection methods: amplitude thresholding, PAPTO, eBOSC, cycle-by-cycle analysis, sliding window matching, and TDE-HMM. The first five methods detect a single beta burst event type and thus can be directly compared in terms of their timing and waveform properties. TDE-HMM, however, identifies multiple spectrally distinct burst patterns not directly comparable with those identified by other methods. In this case, six states are identified and a qualitative summary is provided for illustrative purposes.

Multivariate CDL is applied to whole-head sensor-level data. Similar to TDE-HMM, multivariate CDL is used to extract six spatiotemporal atoms for qualitative analysis. Prior to detection, a beta-band (15–30 Hz) filter was applied to all data to target beta bursts analogous to those detected using the single-channel approaches.

All methods were implemented using open-source python or MATLAB code, available at the links provided in Table 1 and each analysis followed standard methodological practices in the literature. Methods lacking publicly available implementations, or those requiring integration with other tools for burst identification, were not included in this comparison. Runtimes were measured on an Asus workstation (W680-ACE, 2024) running the Ubuntu operating system (v 22.04.5).

**Fig. 2.**
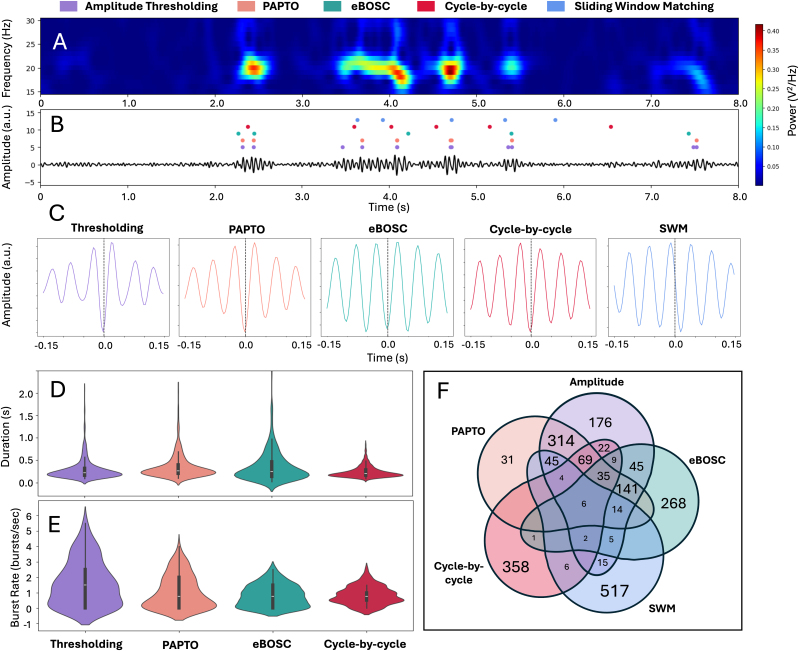
Outputs of single-channel burst detection methods. Results are shown for five methods: amplitude thresholding (purple), PAPTO (orange), eBOSC (green), cycle-by-cycle (red), and sliding window matching (blue). Panel (A) presents a time–frequency decomposition of a representative data segment. Panel (B) shows the bandpass-filtered 15–30 Hz trace for the corresponding data segment with colored dots marking temporal locations of detected events. Panel (C) depicts average waveform shapes aligned to the central trough (Brady & Bardouille, 2022), except for sliding window matching, which returns a representative motif. Panels (D) and (E) display the distributions of event durations and burst rates (computed in sliding 2-second sliding windows). Panel (F) is a Venn diagram summarizing counts and overlaps across methods, illustrating both shared and method-specific detections.

**Fig. 3.**
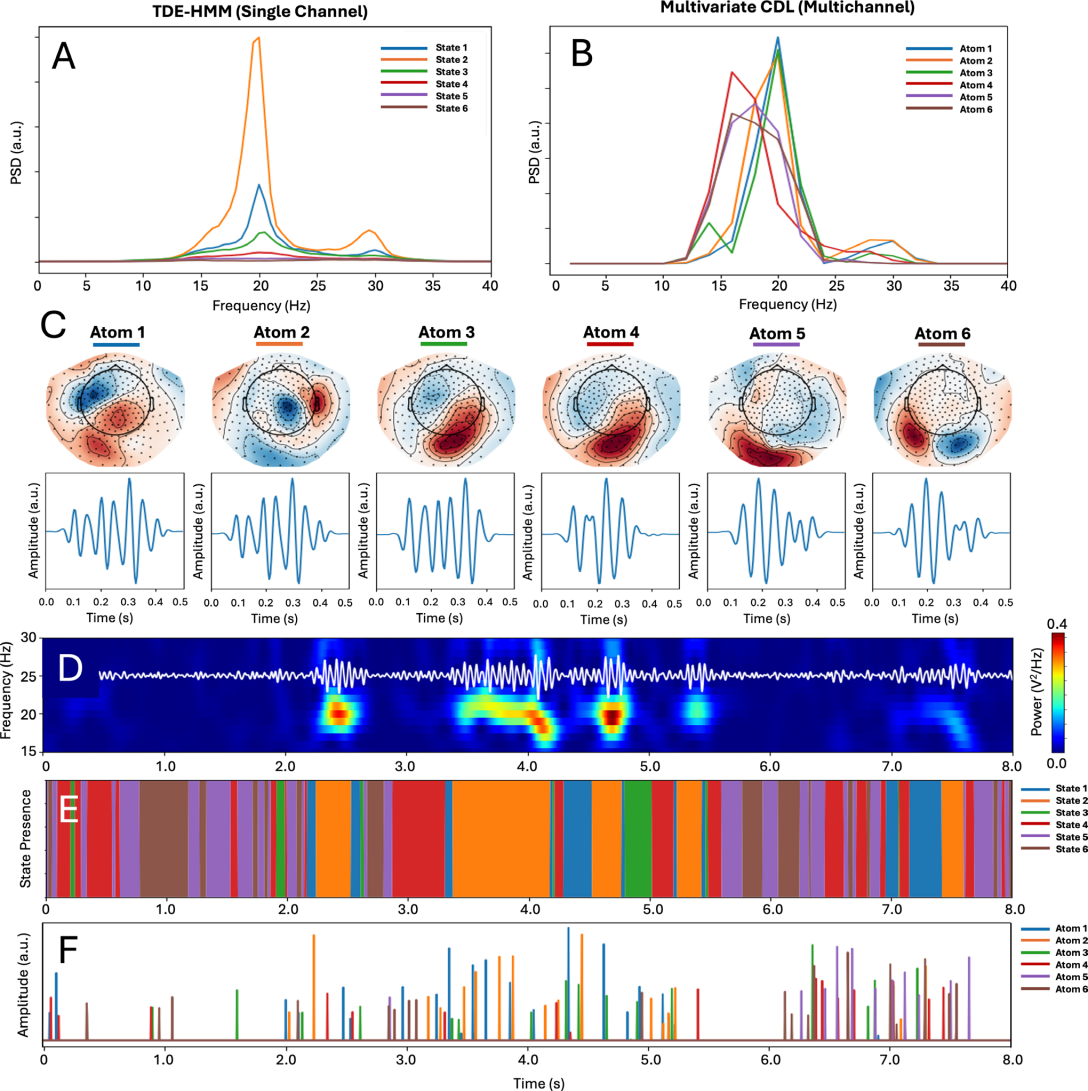
Outputs of motif-based burst detection methods. Model outputs are shown for a 6-state, single channel TDE-HMM decomposition and a 6-atom, whole-head (275 channel) CDL decomposition. Panels A and B present spectral density profiles associated with each of the six HMM states (A) and CDL atoms (B). Panel C further provides illustrative outputs of the distinct spatial (sensor topography) and temporal (motif) profiles associated with each of the multi-channel CDL atoms. Panels D through F show the timing of each of the six HMM states (E) and CDL atom onsets (F) with respect to the raw, right sensorimotor beta-band signal (D), previously presented in Figure 2A and B. Collectively, these outputs highlight complimentary methodological approaches to capturing spectrally distinct events at both single and multi-channel levels.

Figures 2 and 3 present qualitative comparisons of the resulting burst events, spatial maps, motifs, and power spectra. Even when applied to the same dataset, these methods highlight different aspects of transient neural activity: some prioritize sharper waveform transients, others capture broader spatial coordination or state transitions. Their outputs reflect intrinsic trade-offs in temporal precision, spatial coverage, and sensitivity to specific signal features.

### 3.1 Amplitude thresholding

Script:

https://github.com/lindseypower/BurstDetection_Tutorials/blob/main/Tutorials/Amplitude_thresholding.ipynb

Runtime: 0.87 seconds

Amplitude thresholding identifies suprathreshold events in time–frequency space. The procedure begins by defining a power threshold, typically a fixed multiple of the median signal power (e.g., six times the median, as in Shin et al., 2017). A data-driven alternative evaluates a range of candidate thresholds and selects the value that maximizes the correlation between the number of suprathreshold time–frequency points and average signal power; a function implementing this optimization is provided in the tutorial. If a literature-based threshold is adopted a priori, this optimization step can be omitted.

A time–frequency representation (TFR) is then computed by convolving the signal with a Morlet wavelet across the beta band, yielding a time-resolved power estimate at each frequency. High-power events are located by detecting local maxima within the TFR using a regional peak-finding algorithm (e.g., MATLAB’s imregionalmax or a Python equivalent). Peaks that exceed the predefined threshold within the target band are retained as candidate bursts. Each event is subsequently characterized by its time of occurrence, frequency, and peak power, and stored in a structured data frame for analysis.

### 3.2 PAPTO

Script: https://github.com/lindseypower/BurstDetection_Tutorials/blob/main/Tutorials/PAPTO.ipynb

Runtime: 1.42 seconds

PAPTO detects bursts as deviations from a modeled aperiodic background rather than from raw or median-normalized power (Brady & Bardouille, 2022). As in amplitude thresholding, a detection threshold is defined as a multiple of background power, but here the background is estimated from the aperiodic component of the spectrum. Thresholds may be chosen empirically or taken from prior studies; helper functions for threshold optimization are included. Importantly, thresholding is applied to an aperiodic-normalized TFR.

The signal is transformed to the time–frequency domain via Morlet wavelet convolution, identical to the preceding method. The key step is normalization of the TFR using parameters from *specparam* (Donoghue et al., 2020), which fits the log–log spectrum and returns an exponent and offset. These parameters provide a normalization factor that isolates periodic activity from the aperiodic background. Candidate bursts are then identified by local-maxima detection in the normalized TFR, and retained events are summarized by timing, frequency, and power relative to the modeled background.

### 3.3 eBOSC

Script:

https://github.com/lindseypower/BurstDetection_Tutorials/blob/main/Tutorials/eBOSC.ipynb

Runtime: 59.70 seconds

eBOSC extends the BOSC framework by modeling the aperiodic spectrum with robust regression and by offering additional options for event characterization (Kosciessa et al., 2020). Bursts are defined as time–frequency segments that exceed both a power threshold and a minimum duration, relative to the fitted background spectrum. Model parameters (frequency range(s), minimum duration in cycles, and post-processing options such as event merging) are specified in a configuration dictionary. Defaults are provided, but settings should be adapted to the dataset and research aims, following the guidance in the eBOSC documentation.

Detection is executed via a single call to the package wrapper, which computes the TFR, estimates the background spectrum in log–log space using robust regression, and applies the joint power-and-duration criteria. The wrapper returns a structured object containing maps of detected events, the fitted background spectrum, and summary statistics for each burst. These outputs can be queried or visualized directly and integrated with downstream analyses.

### 3.4 Cycle-by-cycle analysis

Script:

http://github.com/lindseypower/BurstDetection_Tutorials/blob/main/Tutorials/Cyclebycycle.ipynb

Runtime: 1.73 seconds

Cycle-by-Cycle analysis defines bursts by waveform shape and rhythmic consistency rather than power thresholds (Cole & Voytek, 2019). The approach first specifies thresholds for cycle-level features: amplitude and period consistency relative to neighboring cycles, rise–decay symmetry, and the minimum number of consecutive qualifying cycles required to constitute an event. Example settings suitable for beta activity are provided in the tutorial and can be adjusted to balance sensitivity and specificity.

After band-limited filtering, detection is performed with the Bycycle Python package, which returns a data frame containing cycle timings and features, together with an is_burst label for cycles meeting the criteria. Consecutive burst-labeled cycles are then grouped post hoc, using peak and trough timings to define event boundaries. From these segments, event-level measures (duration, mean amplitude, and peak frequency) are derived, facilitating comparison with threshold-based methods.

### 3.5 Sliding window matching

Script: https://github.com/lindseypower/BurstDetection_Tutorials/blob/main/Tutorials/Sliding_window_matching.ipynb

Runtime: 11.06 seconds

Sliding window matching detects recurring waveform motifs by scanning the signal with a fixed-length window and searching for repeated patterns (Gips et al., 2017). Two parameters need to be set: the window length and the number of motifs to extract. The window length sets the dominant temporal scale (longer windows bias toward slower rhythms) and should approximate the expected burst duration; the number of motifs reflects an initial estimate of distinct burst types.

Using utility functions provided in the repository, a Monte-Carlo search samples candidate windows and identifies motifs that recur throughout the recording. The output includes representative motifs and the time points at which each motif appears, enabling subsequent event characterization

As illustrated in Figure 2, outcomes reflect each method’s implicit definition of a burst. Amplitude-based procedures (amplitude thresholding, PAPTO, eBOSC) favor high-power transients, whereas waveform-centric procedures (Cycle-by-Cycle, Sliding Window Matching) often capture lower-power but shape-consistent events. Methods that use Morlet transforms tend to select wavelet-like waveforms, while others admit oscillations that are flatter or more symmetric. Simpler algorithms generally yield more detections; more elaborate models emphasize specificity. Overlap is greatest among methods that share similar computational assumptions.

### 3.6 Time-delay embedding hidden Markov modeling (TDE-HMM)

Script:

https://github.com/lindseypower/BurstDetection_Tutorials/tree/main/Tutorials

Runtime: 16.32 seconds

Prior to training, time-delay embedding is applied (via the prepare routine). Model hyperparameters are set in a Config object, including the number of states and computational settings (sequence length, batch size, learning rate, epochs), selected according to the recommended default values for the model. The model is instantiated and training proceeds with the fit procedure, yielding recurrent states in the embedded space. State probability time courses are then used to segment the original data, from which state-specific power spectral densities and state time courses are computed and visualized.

### 3.7 Convolutional dictionary learning (CDL)

Script:

https://github.com/lindseypower/BurstDetection_Tutorials/blob/main/Tutorials/CDL.ipynb

Runtime: 204.62 seconds

CDL requires specification of a pattern duration and the number of patterns to extract. For burst detection, the duration is typically set to approximately twice the expected event length to capture the full motif. The algorithm (implemented via repository utilities alongside open-source packages; Dupré la Tour et al., 2018; Jas et al., 2017) performs convolutional sparse coding to learn temporal atoms, their sensor-space topographies, and activation vectors. Activation vectors provide timing and relative power of associated events.

Both the single-channel HMM and multichannel CDL approaches segment the data into multiple spectrally distinct motifs. While purely data driven in their approaches, the example shown in Figure 3 demonstrates that both methods identify at least one motif for which the activation aligns with high-power beta bursts visible in the raw, sensorimotor signal. State 2, identified by the TDE-HMM approach, reflects high-power beta activity (Fig. 3A) and has a state time course that aligns temporally with high-power events in the raw signal (Fig. 3D, E). Similarly, CDL atom 2 reflects high beta power (Fig. 3B), a right central spatial pattern typical of right sensorimotor activity (Fig. 3C), and an activation time course (Fig. 3F) that approximately aligns with the onset of high-power events in the raw signal. The additional utility of these methods for simultaneously detecting other events of interest is also highlighted in Figure 3, with HMM highlighting several spectrally distinct states that could reflect lower power burst subtypes and CDL identifying other high-power bursting events with distinct spatial profiles and waveform shapes.

## 4 Guidelines for Method Selection

As demonstrated throughout this review, the methodological landscape for burst detection is both expansive and nuanced. Selecting an appropriate approach for a given research or clinical application requires careful consideration of factors including signal characteristics, analytic goals, and computational constraints. Before initiating a burst-based analysis of electrophysiological data, it is essential to reflect on the spatial specificity of the research question, the desired characterization of oscillatory activity, and the extent to which assumptions about waveform shape or spectral content are acceptable. The importance of spatial information should also be considered, as should the balance between hypothesis-driven constraints and the flexibility of data-driven exploration.

One important consideration is the type of neurophysiology data that is available. Multi-channel approaches have been primarily validated for whole-head, non-invasive recordings such as MEG or EEG, whereas invasive multichannel data such as intracranial EEG grids currently lack the same level of validation. In these invasive cases, single-channel approaches remain the standard. For whole-head data, the choice can be further guided by determining whether a clear spatial hypothesis exists regarding the signal origin, and whether the analysis benefits from explicitly modeling relationships between neighboring channels. In the absence of strong spatial constraints, multi-channel methods may be preferable, whereas single-channel approaches often yield more direct and interpretable characterizations of bursts.

For single-channel approaches, it is also important to define what constitutes a burst in the context of the study. Amplitude-based methods such as amplitude thresholding, PAPTO, and eBOSC typically define bursts by amplitude criteria, making them well suited for detecting high-power events and straightforward to implement. Waveform and motif-based methods, by contrast, prioritize waveform morphology. Waveform template matching motif-based approaches aim to identify repeating patterns, while cycle-by-cycle analysis isolates bursts based on consistent amplitude and periodicity across cycles. Clearly articulating which burst features (amplitude, periodicity, waveform shape, or duration) are most relevant is critical for method selection. As illustrated in Figure 3, methods with similar burst definitions tend to produce overlapping results, with amplitude-based techniques agreeing most strongly with each other and diverging from waveform and motif-based methods.

Practical considerations also play a role. Computational cost, implementation complexity, and software availability can be decisive factors. For example, there are considerable differences in the runtimes of various methods, with more computationally complex methods typically requiring longer computation times. Approaches such as eBOSC and multivariate CDL have particularly long runtimes and may demand substantial computational resources, while amplitude thresholding, PAPTO, and Cycle-by-Cycle analysis are faster and relatively lightweight. This consideration is particularly relevant when processing large amounts of data or in the case of single-channel methods, when repeating the detection analysis across many channels or brain regions. Table 1 summarizes empirical applications, software tools, and reference materials to assist with evaluating these trade-offs.

Regardless of the chosen approach, users should review the method’s assumptions and configurable parameters (see Table 1), as these can substantially influence results. Settings such as thresholds, motif durations, or window lengths must be tailored to the dataset and research objectives. Parameter choices in preprocessing steps such as filtering parameters and frequency transform methods (Fourier, wavelet, superlet, etc.) may also impact burst outputs and should be carefully considered when designing an analysis pipeline (see review by Cho & Choi, 2023). Critically, the user should evaluate the appropriateness of their methodological choices at each stage of analysis, by assessing model fits (e.g., aperiodic fit when using specparam, explained variance, or goodness of fit metrics when using signal decomposition approaches) and plotting burst outputs with respect to raw signals (e.g., Fig. 2). Consulting tutorials and documentation, such as those linked throughout this review, is strongly recommended to ensure that parameter choices are justified, and that the interpretation of results is methodologically sound.

## 5 Conclusion

Neural burst detection has become a powerful analytical approach for studying electrophysiological signals, driving substantial methodological progress over the past decade. This progress has produced an expanding array of techniques for both single- and multi-channel data, each grounded in distinct operational definitions and assumptions about what constitutes a burst. In this diverse methodological landscape, it is essential to understand the scope, strengths, and limitations of each approach to ensure that the chosen method is both scientifically justified and technically sound.

The field continues to evolve rapidly, with emerging technologies offering promising avenues for further innovation. Artificial intelligence and deep learning are increasingly being integrated into data-driven burst detection, particularly in whole-brain MEG/EEG and high-density intracranial recordings. On the hypothesis-driven side, optimization algorithms are beginning to provide more rigorous and standardized approaches to hyperparameter selection, improving reproducibility while accommodating dataset-specific characteristics.

In such a dynamic context, informed methodological choices require careful consideration of model inputs and outputs, the validity of underlying assumptions, and practical factors such as data type, computational constraints, and the specificity of research hypotheses. Aligning these considerations with study objectives will help ensure transparent, reproducible, and conceptually coherent research. By doing so, investigators can more effectively harness burst detection to advance our understanding of the transient and dynamic nature of brain activity, and the neural signatures that underpin cognition, behavior, and disease.

## Data Availability

Data used in this work are freely available at: https://openneuro.org/datasets/ds000247/versions/00001. Tutorial code is available at: https://github.com/lindseypower/BurstDetection_Tutorials.
